# Drug-Induced Immune Thrombocytopenia From Administration of a Local Anesthetic Agent Resulting in Splenectomy

**DOI:** 10.7759/cureus.8293

**Published:** 2020-05-26

**Authors:** Safwan Muhammad, Ammad Naeem, Amna Shaukat, Subas Javaid, Saqib Alvi

**Affiliations:** 1 Internal Medicine, University of Maryland Medical Center Midtown Campus, Baltimore, USA; 2 Internal Medicine, University at Buffalo, Buffalo, USA; 3 Internal Medicine, Services Institute of Medical Sciences, Lahore, PAK; 4 Internal Medicine, Liaquat National Medical College, Karachi, PAK; 5 Pathology, Penn State Health Milton S. Hershey Medical Center, Hershey, USA

**Keywords:** drug-induced immune thrombocytopenia, local anesthetic, sodium channel blocker, splenectomy

## Abstract

Thrombocytopenia is a common clinical condition, and drug-induced immune thrombocytopenia (DITP) should be considered in hospitalized patients with severe thrombocytopenia who are exposed to new medications. The potential mechanism is described to be drug-triggered antibody-mediated platelet destruction causing petechiae and mucosal bleeding. Severe form of DITP can be refractory to systemic steroids and even intravenous immunoglobulin administration. Such cases usually require splenectomy for definitive treatment. A number of substances including medications, herbal remedies, and even food items have been identified with a definitive or probable causal role in DITP. However, it is rarely reported from locally administered medications such as local anesthetic drugs. We present a unique case of severe DITP from lidocaine that resulted in refractory DITP requiring splenectomy for definitive treatment.

## Introduction

Drug-induced immune thrombocytopenia (DITP) is caused by the rapid destruction of platelets due to drug-dependent, platelet-reactive antibodies [1-3]. The proposed mechanism involves antiplatelet antibodies binding non-covalently to the specific platelet antigens. Splenic macrophages act as antigen-presenting cells [4]. DITP and immune-mediated thrombocytopenia are similar in presentation since patients are otherwise healthy except for low platelet count and/or bleeding; however, DITP is triggered by a drug or substance. DITP also tends to resolve with the removal of offending agents in most cases. We present a unique case of severe recurrent DITP after exposure to a local anesthetic agent, such as lidocaine, that led to petechiae and hematuria, which eventually required splenectomy for definitive treatment.

## Case presentation

**Figure 1:**
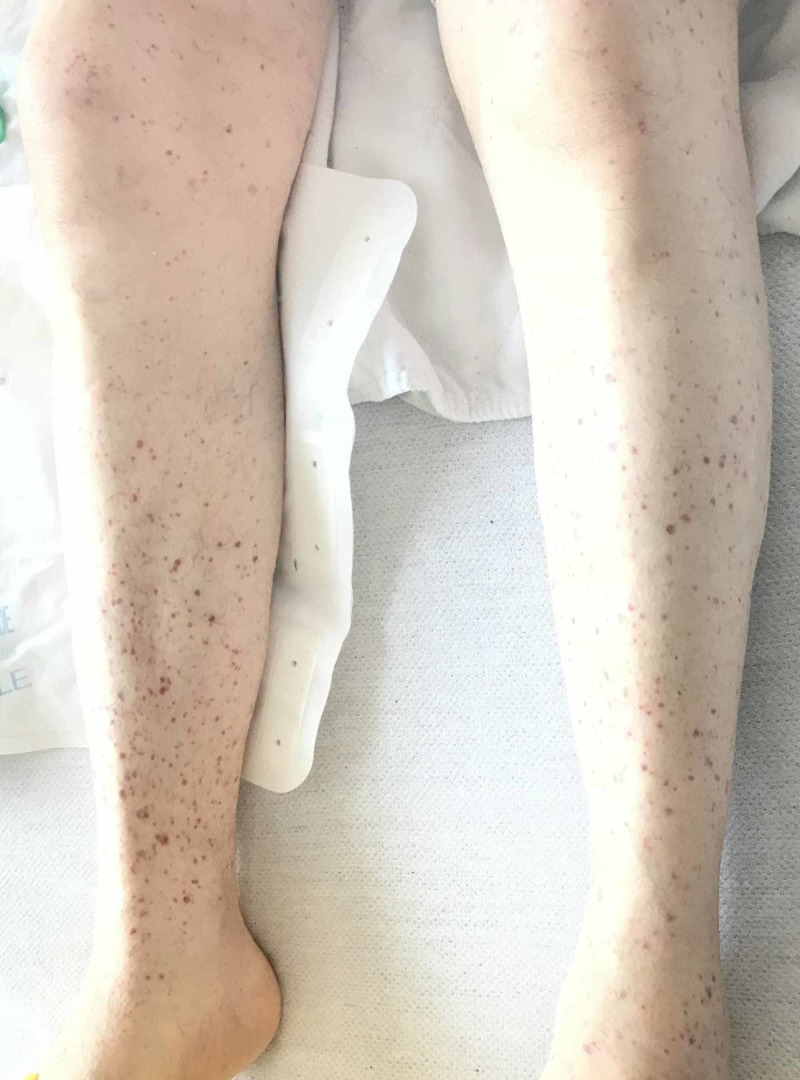
Petechiae on both shins

**Figure 2:**
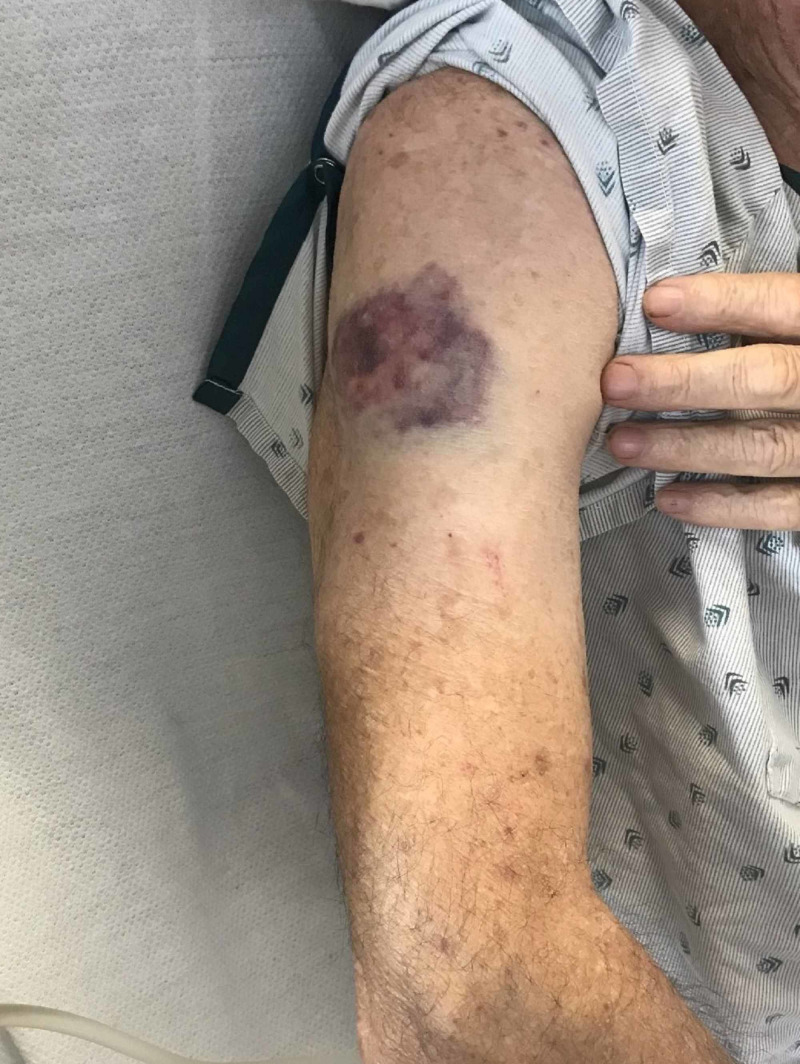
Bruise on the right arm

A 75-year-old white male with a history of pre-diabetes, benign prostatic hyperplasia, hypertension, and hyperlipidemia presented with severe thrombocytopenia following a dental extraction where he was administered local anesthetic lidocaine preoperatively. His preoperative platelet count a week before the procedure was 160,000 per microliter. His routine home medications included a statin and a calcium channel blocker for hypertension. He denied using any new drug in the past several months. He received no antibiotic prophylaxis prior to dental extraction. Shortly after the administration of the drug, the patient developed petechiae over his extremities and bruising (Figures 1, 2). 

In addition, he developed epistaxis and gross hematuria, which prompted him to come to the emergency department for further evaluation. On presentation, the patient's platelet count was 9,000 per microliter. He was transfused multiple units of platelets which remained ineffective in stopping hematuria, and he had no improvement in his platelet count. The patient also failed a course of intravenous steroids. A course of intravenous immunoglobulins (IVIG) was given to no avail which made a diagnosis of DITP more likely. Hematuria did not resolve and platelet count remained consistently below 10,000 per microliter despite the above interventions. After consultation with the hematologist, it was decided that splenectomy is warranted at this point. Two days after the splenectomy, the patient’s platelet count increased to 111,000 per microliter. Upon further investigation, the patient reported that he was diagnosed with immune thrombocytopenia two years ago when he developed a petechial rash with a platelet count of 3,000 per microliter after he received a spinal injection for his chronic back pain. Anesthetic used at the time was also lidocaine. Records indicated that at that time, the patient did not respond to steroids completely and was treated with IVIG. He was later discharged on a steroid taper; however, he stopped the taper prematurely and ended up receiving IVIG for a relapse. 

## Discussion

DITP is a form of secondary immune thrombocytopenia, which is caused by drug-dependent antibody-mediated platelet destruction. Commonly implicated drugs in this condition are quinine, mirtazapine, trimethoprim-sulfamethoxazole, penicillin, and carbamazepine. A formal database of offending drugs is updated biennially. The list of such drugs usually expands by one of the three ways, i.e. case reports with definitive evidence, identification of new drug-dependent antibodies, and from data mining of United States Food and Drug administration's adverse events reporting system database [5].

A thorough literature review did not reveal any case report of DITP after exposure to local anesthetic medication. This case presents a patient who developed secondary immune thrombocytopenia following an initial exposure and then severe refractory DITP after rechallenge two years later with a local anesthetic, which acts as a sodium channel blocker. DITP usually results in a severe decline in platelet count and patients can have a nadir platelet count of less than 2,000 per microliter [6,7]. Our case experienced platelet count as low as 9,000 per microliter. This is different from heparin-induced thrombocytopenia in which the platelet count remains more than 60,000 per microliter.

Intentional rechallenge of the drug is generally not advised due to high risk of recurrence; however, rechallenge may occur inadvertently as happened in this case who was initially labeled as immune-mediated thrombocytopenia without any confirmed secondary etiology. Interestingly, rechallenge may provide definitive evidence that the immune-mediated thrombocytopenia is indeed secondary to the drug-induced mechanism such as in our case where the patient had a severe recurrence and platelet count dropped to as low as 9,000 per microliter.

As mentioned earlier, several drugs are implicated in a similar phenomenon which can provide useful insight into patient care in such cases. For example, a prospective review of 550 patients who received rechallenge with glycoprotein IIb/IIIa inhibitor abciximab at least one week apart demonstrated severe DITP in 2.4% and more interestingly 0.4% patients developed it after discharge [8]. Another study argued that the duration between the first dose and rechallenge dose may increase the incidence of recurrence of severe thrombocytopenia from 4% to 12% if rechallenge occurs in less than two weeks [9]. These findings are especially important for patients who may be at risk of bleeding after the discharge and may have morbidity related to this condition.

## Conclusions

Severe thrombocytopenia resulting in an acute setting should prompt a review of new medications that may be involved in antibody-mediated destruction of platelets. Since DITP can potentially result in bleeding, providers should update the patient records to document such events to avoid inadvertent rechallenging. Providing medical alert bracelets to avoid such events may also be beneficial. 
